# Kentucky Medical Society

**Published:** 1883-05-20

**Authors:** T. B. Greenley


### Southern Medical Record:

EDITORS:

T. S. POWELL, M.D. W. T. GOLDSMITH, M.D. R. C. WORD, M.D.

*R. C. WORD, M.D., Managing Editor.*

☞ All Communications and Letters on Business connected with the RECORD must be addressed to the Managing Editor.

VOL. XIII. ATLANTA, GA., MAY 20, 1883. NO. 5.

### ORIGINAL AND SELECTED ARTICLES.

### KENTUCKY MEDICAL SOCIETY.

*Editors Southern Medical Record:*

I have just returned from the annual meeting of our State Medical Society. It convened on the 4th instant, and continued three days.

We had a fine attendance, and a very good meeting; a great many papers of interest being read.

Our President, Dr. Price, delivered quite an able address, touching particularly the subjects of Medical Education and Medical Ethics. These are, at this time, apparently, objects of much interest.

The report on Medical Ethics, by Dr. Yager, was quite able, and elicited much interest on the part of members. He is greatly opposed to the recent revolution on the part of the State Society of New York, and criticised its action in quite positive language. It would seem, however, that that body, at least a majority so far, still clings to its ideal with much tenacity. But to judge of the tone of its former advocates and supporters, I would not be surprised if the project was not abandoned before the lapse of many years. The irregulars, with whom it has seemed of late so desirable to connect, it is said, intend to have their own specialties, and thereby save the cases to their own side of the house. This plan would leave our new-code men out in the cold.

The report of the committee on Surgery, by Dr. Fuqua, was very able, and presented the improvements and advances in that department in quite a terse and interesting manner.

Dr. Scott, of Louisville, reported on Obstetrics. He dwelt particularly on the want of due attention given by mothers to daughters, respecting their menstrual functions. His argument led to the point that, on account of neglect in this particular, the exigencies of the child-bearing period were greatly enhanced. Also alluded to the pernicious effects of being over-worked during the access and continuance of the mestrual flow. That this is a time when the economy requires, and should receive, comparative rest. There can be little doubt that over muscular exercise at this period tends greatly to break down nervous power, and thereby materially weaken the reproductive organs. I regard his report as being quite valuable.

Dr. Ochterlony reported on Dermatology, which was one of the best papers presented. He spoke at length of the great advantage of the use of hot water applications in the treatment of that terribly distressing disease, prurigo. It is better, if convenient, to immerse the parts involved in as hot water as can well be borne, for several minutes at a time, and well wiped dry after each immersion.

The report on Materia Medica was made by your humble servant, but modesty forbids favorable criticism. Of course, on the other hand, I would not like to speak disparagingly ot my own work.

There were papers read on "The Vagaries of Medicine," by Dr. Speed; "Clinical Observations on Head Injuries," by Dr. Roberts; "A Case of Strichnia Poisoning," by Dr. Seargent; "Two Orthopedic Cases," by Dr. Vance; "On Tracheotomy," by Dr. Wilson; "On Pertussis," by Dr. Webb; "On Conjunctival Blenorrhœa," by Dr. Ferguson. Dr. Rumbold, by invitation, read a paper on the treatment of "Chronic Naso-Pharyngeal Catarrh."

Your humble servant read a paper on "Fecal impaction of upper portion of sigmoid flexure of colon, accompanied by persistent diarrhœa."

Many of these papers were quite able, and elicited much interest.

The paper of Dr. Speed, on the "Vagaries of Medicine," criticised the character of Hahnemann with great severity, and proved him to have been the merest charlatan. His criticisms of the "Similia Similibus Curanter" dogma, as well as the infinitesimal absurdity, were extremely caustic in their severity. The Doctor is well posted in what is termed homeopathy. He regards it as the quintessence of absurdity.

The remarks of Dr. Roberts on Head Injuries were very interesting as well as instructive. He had relieved several patients of epilepsy by trephining and removing the cause. His report of cases were corroborated by the remarks of Prof. Yandell, with illustrative cases.

The case of strychnia poisoning, by Dr. Seargent, was one of great interest. The patient had taken twenty grains of the alkaloid one hour before the Doctor saw him. He was treated with chloral hydrate and bromide of potassium successfully. None of the poison was rejected by the stomach. It was regarded by the Society as a remarkable cure.

Dr. Vance illustrated his cases of orthopedic deformities, as well as the cures, by photograph drawings. The Doctor, though quite young, is becoming to be quite an expert in that variety of surgery.

Doctor Wilson performed tracheotomy on a child for diphtheria, which proved successful, the only one for that disease in the city of Louisville. He was complimented on his success by Professor D. W. Yandell.

Dr. Ferguson entertained us very ably in discussing conjunctival blenorrhœa and its treatment, as observed in the Royal Ophthalmic Hospital of London. He described the nature and character of the disease very accurately, and made cleanliness a great point in the treatment.

Dr. Rumbold's plan of treatment for chronic catarrh of the nose and throat is simple, and he says quite effective. He, by atomizing tubes, applies melted vaseline to the parts affected. He claims for this article virtues superior to all others. His apparatus, however, I would judge from appearances, to be quite expensive.

Dr. Wilson exhibited an improved hollow needle, curved for the purpose of closing openings in the vagina, either rectal or cystic. It has many advantages over those in use, both as regards size and length. Of course it is only adapted to the use of wire ligatures.

Upon the whole, our meeting was a success, more so as it respects ability of papers presented than as to numbers. The weather during the session was rather unpropitious, which perhaps kept some away who otherwise would have been present.

Two years ago, it was resolved by the Society that Louisville should, in the future, be the permanent place of holding our annual meetings. But at this meeting we have agreed to re-inaugurate the old plan of a migratory Society. We shall, I suppose, hereafter meet wherever we may be invited to come. I think, perhaps, this last change was brought about by an apparent dissatisfaction on the part of the provincials at the metropolitans for seemingly wishing to assume the privilege of keeping a *good* thing too much to themselves.

We shall hold our next meeting at Bowling Green, on the first Wednesday of May, next year, provided it does not conflict with the meeting of the American Medical Association. In such event a timely notice will be given.

Dr. McCormack, of Bowling Green, was elected our next president. Yours very truly,

T. B. GREENLEY.

P. S.—Hope Georgia will be largely represented at Cleveland in June. Be glad to see you and Dr. Powell present.

Yours, etc., G.

